# Genetic Mapping and Characterization of Verticillium Wilt Resistance in a Recombinant Inbred Population of Upland Cotton

**DOI:** 10.3390/ijms25042439

**Published:** 2024-02-19

**Authors:** Iain W. Wilson, Philippe Moncuquet, Yuman Yuan, Melanie Soliveres, Zitong Li, Warwick Stiller, Qian-Hao Zhu

**Affiliations:** 1 CSIRO Agriculture and Food, GPO Box 1700, Canberra, ACT 2061, Australia; 2 CSIRO Agriculture and Food, Locked Bag 59, Narrabri, NSW 2390, Australia

**Keywords:** verticillium wilt, disease resistance, *Gossypium hirsutum*, *Verticillium dahliae*, cotton, recombinant inbred lines, genomic prediction

## Abstract

Verticillium wilt (VW) is an important and widespread disease of cotton and once established is long-lived and difficult to manage. In Australia, the non-defoliating pathotype of *Verticillium dahliae* is the most common, and extremely virulent. Breeding cotton varieties with increased VW resistance is the most economical and effective method of controlling this disease and is greatly aided by understanding the genetics of resistance. This study aimed to investigate VW resistance in 240 F_7_ recombinant inbred lines (RIL) derived from a cross between MCU-5, which has good resistance, and Siokra 1–4, which is susceptible. Using a controlled environment bioassay, we found that resistance based on plant survival or shoot biomass was complex but with major contributions from chromosomes D03 and D09, with genomic prediction analysis estimating a prediction accuracy of 0.73 based on survival scores compared to 0.36 for shoot biomass. Transcriptome analysis of MCU-5 and Siokra 1–4 roots uninfected or infected with *V. dahliae* revealed that the two cultivars displayed very different root transcriptomes and responded differently to *V. dahliae* infection. Ninety-nine differentially expressed genes were located in the two mapped resistance regions and so are potential candidates for further identifying the genes responsible for VW resistance.

## 1. Introduction

Verticillium wilt (VW) is an important disease of cotton with the causative agent being the soilborne hemibiotrophic fungus *Verticillium dahliae* Kleb [1]. The fungus invades through the roots and once in the xylem produces conidiospores that spread acropetally throughout the plant [1]. During infection, the *V. dahliae* secretome supplies a range of molecules, such as toxins, to manipulate the host responses and aid its growth that can result in vascular occlusion, which prevents the transfer of water and other mineral substances from roots to the leaves and tissues and causes wilting, drying, a reduction in photosynthesis, shedding of immature bolls, and importantly a significant reduction in fiber yield [2,3,4]. In the field, the disease is characteristically associated with vascular discoloration, leaf chlorosis, necrosis, and plant death in severe cases. Once cotton tissues become necrotic, the fungus produces highly melanised resting structures called microsclerotia which are released in the soil upon plant decomposition and can remain viable in the soil for nearly 10 years [5]. VW is considered a polyetic disease, as inoculum can increase in field soils from one season to the next which can result in a progressive increase in VW incidence and severity over succeeding years [6]. So, once the disease is established, it is nearly impossible to eliminate and difficult to manage.

Classification of strains of *V. dahliae* in cotton has been traditionally based on the symptoms exhibited by the host plant, vegetative compatibility groups (VCG) based on complementation with auxotrophic nitrate-non-utilizing mutants (VCG 1, 2, and 4, which can further be subdivided into A and B in cotton) [7,8], or by the presence or absence of the *Ave1* virulence gene (race 1 and 2) [9]. Strains in VCG 1 are the defoliating (D) pathotype and belong to race 2, while those in VCG 2 and VCG4 groups are non-defoliating (ND) pathotypes and belong to race 1. Sequence data from intergenic spacer regions can provide presumptive VCG identification [10], and a PCR-based test can discriminate between D and ND [11] pathotypes. However, the availability of *V. dahliae* genome sequences [12,13] has indicated that this species is not amenable to facile classification. In Australia, it was previously thought that only ND VCG4B was present in cotton soils, but in 2014 the presence of ND VCG2A was confirmed [14] and recently VCG1A was found [15]. However, unlike most cotton-growing countries, not only is ND VCG2A *V. dahliae* the most prevalent pathotype in Australian cotton fields, but it also has the ability to cause severe defoliation and crop losses comparable to that caused by VCG1A [6,16]. However, the virulence of any specific Australian isolate is not determined by its VCG and requires infection-based validation [17].

The life cycle of *V. dahliae* makes managing the disease difficult, requiring a fully integrated disease management strategy that focuses on first preventing the spread of the disease (Come Clean Go Clean [18]) and then on limiting fungal inoculum levels building up in the soil. Currently, this is performed through a combination of soil fumigation [19], fungicide seed coatings, long crop rotations with managing weeds that are a potential host for *V. dahliae* [20,21,22], and incorporating crop residues into the soil as soon as possible after harvest. But long term, the most practical solution is the development of resistant cotton varieties [23,24]. In Australia, the development of varieties with increasing resistance to VW started with the release of the Upland cultivars Sicala V-1 in 1990 and Sicala V-2 in 1994 [25]. The level of resistance of these cultivars has been essentially maintained, as much of the resistance found in current commercial cultivars is derived from these resistant cultivars [23]. However, despite maintaining relatively high VW resistance levels by international standards [25], the incidence of VW has continued to rise over the last decades [26]. The discovery of additional *V. dahliae* pathotypes in Australia has made breeding for VW more difficult as recent observations suggest that resistance to one pathotype is not necessarily associated with resistance to another, requiring that ND and D *V. dahliae* pathotypes are treated essentially as independent breeding targets [23].

Studies on the inheritance of VW resistance have been somewhat contradictory, with studies reporting relatively simple inheritance, based on one or few major genes [27,28,29], whereas others report resistance as a quantitative trait [30,31,32,33,34,35,36]. These differences appear to be largely dependent on the observation that the disease severity is highly dependent on environmental conditions, with severe disease prevalent in cooler, wet, and humid environments as well as excessive soil nitrogen and deficiency of potassium [37]. Although Australian cotton cultivars have relatively high levels of VW resistance, they are known to become more susceptible to VW disease when soil temperatures drop below 22 °C [6]. Also, in general, methods for assessing VW resistance are visually based and operator-dependent, which may contribute to the lack of correlative inheritance determinations between studies [30]. Other variables that affect VW studies include cotton species tested, *V. dahliae* pathotype and isolate used, whether controlled conditions or the field environment is used for infection, and the developmental stage (seedling versus adult) at which plants are assessed for resistance [38]. Currently, there are no studies associated with the inheritance of VW resistance with cotton varieties infected with Australian *V. dahliae* isolates.

This study aimed to determine the inheritance of VW resistance to a virulent ND VCG2A *V. dahliae* in 240 F_7_ recombinant inbred lines (RIL), with the objective of discovering potential candidate genes associated with VW resistance that could be deployed in a breeding program.

## 2. Results

### 2.1. Segregation of VW Resistance in F_7_ RIL Lines

To minimize many of the environmental variables that affect studying VW resistance and to attempt to identify VW resistance effective at low temperatures, our research was based on 240 F_7_ RILs assayed for VW resistance under controlled conditions in a greenhouse where temperatures were maintained at ~22 °C. The advantage of RILs is that by the F_7_ generation, each line is close to being genetically fixed, so experiments can be replicated with near-identical genetic background seedlings from the same line. In total, thirty plants (in two separate experiments) from each of the 240 lines were infected (*4.2 V. dahliae Pathotype*, *Inoculum Preparation*, and *Inoculation System*) with the same inoculum dose from a pure ND VCG 2A, Race 1 *V. dahliae* isolate that was recovered from a cotton plant in the field displaying severe VW symptoms. Infected plants were compared to an identical number of plants from the same line that underwent a mock infection at the same time, and resistance was measured based on relative survival and fresh shoot weight of lines between infected and mock-infected after four weeks (Supplementary Table S1).

**Figure 1:**
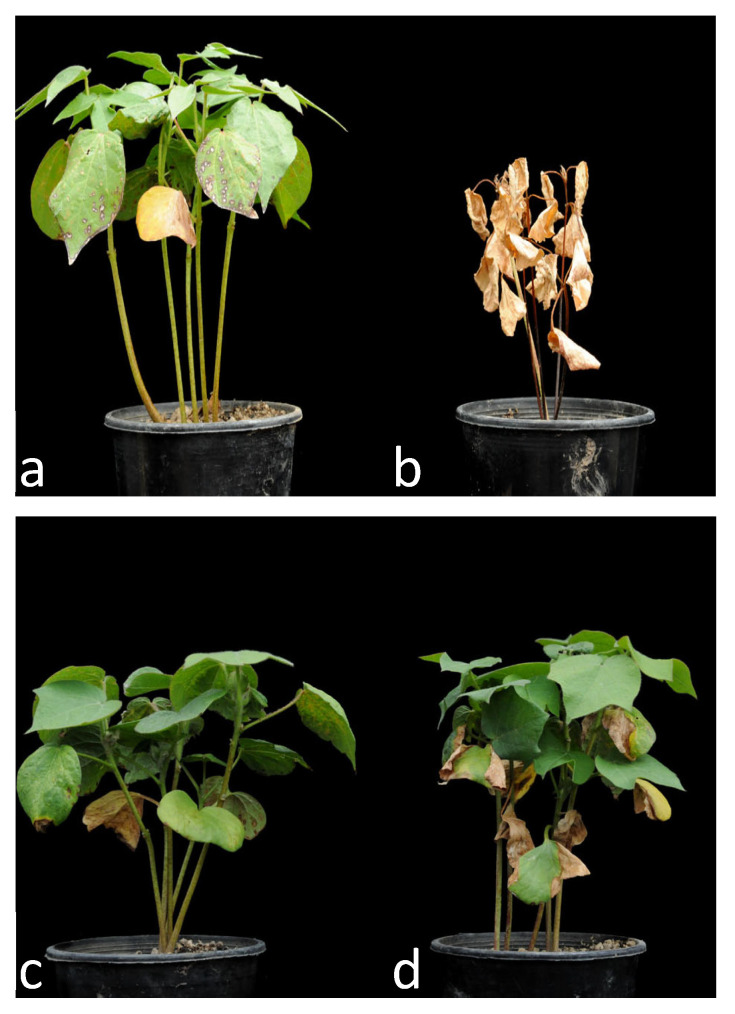
Verticillium wilt resistance of Siokra 1–4 uninfected (**a**) and infected (**b**) and MCU-5 uninfected (**c**) and infected (**d**).

The two parent lines used to construct the RIL population as expected displayed contrasting levels of resistance to the ND VW pathogen (Figure 1). On average, MCU-5 plants survived 92% (SD = 8) and had a relative shoot fresh weight of 56% (SD = 21), whereas Siokra 1–4 plants survived only 5% (SD = 7) and had a shoot fresh weight of 19% (SD = 21). The distribution of relative survival in the RIL population (Supplementary Figure S1) revealed a slightly bi-modal appearance but the majority of the lines were susceptible to infection with 147/240 lines displaying ≤30% survival, whereas only 13/240 lines had survival >80%. The shoot fresh weight scores for the population (Supplementary Figure S2) also indicated that most lines’ growth was affected by the infection with 152/240 having fresh weight of ≤30% compared to their uninfected controls, and no lines had similar shoot weights (90–100%) to mock-infected controls.

### 2.2. QTL and Genomic Prediction Analysis of VW Resistance

A total of 1337 genetic markers consisting of mostly Illumina GoldenGate SNPs data and simple sequence repeat (SSR) markers were genotyped on the 240 F_7_ RILs derived from MCU-5 x Siokra 1–4, with the mapping data previously published [39]. GoldenGate SNP markers were named according to their location on the genome of *G. raimondii* [40]. The linkage disequilibrium (LD) network approach was used to cluster the markers into 461 LD blocks. Two QTL mapping methods were applied: a single-locus mapping using linear regression and permutation test [41], and multiple-locus mapping using a modified Bayesian stochastic search variable selection algorithm [42]. The single-locus approach identified twelve and eighteen significant markers (*p*-value < 0.05) associated with shoot weight and survival, respectively, with markers located in ChrD03 (LD blocks 205, 206, 207) and ChrD09 (LD block 333) (Table 1 and Table 2).

The multiple-locus approach identified two significant markers for both weight and survival, from the same ChrD03 and ChrD09 genomic regions as detected in single-locus mapping (Table 3) (i.e., selection probability >0.5). The genomic heritability for survival based on the multiple-locus analysis was found to be 0.58, dominated by just two markers: Chr03_2558470 and Chr06_48100923, which explained 0.37 and 0.20, respectively, of the phenotypic variation. Shoot weight was found to have lower genomic heritability (0.35) controlled by the same two markers (Chr03_2558470 and Chr06_48100923) but explaining less of the phenotypic variation (0.14 and 0.06, respectively).

An important metric for breeding for increased resistance is the prediction accuracy of selecting a line based on their genotype alone. To determine the predictive power, the multiple-locus method on the shoot weight and survival traits was evaluated using a 5-fold cross-validation strategy. The genomic prediction accuracies were 0.36 (SE = 0.04) and 0.73 (SE = 0.03) for shoot weight and survival, respectively.

### 2.3. Comparative Transcriptome Analysis of MCU-5 and Sikra 1–4 to V. dahliae Infection

To explore the gene expression changes associated with the response to *V. dahliae* infection in resistant MCU-5 and susceptible Siokra 1–4, RNA-seq analysis was performed on root tissue taken from uninfected (0 h-post-infection, hpi) and *V. dahliae* infected and mock-treated plants at 6 hpi, 1 and 3 days-post infection (dpi). On average, each of the 42 samples sequenced had over 27.6 million clean paired-end reads, and 62–77% (average = 71%) of clean reads from these samples could be aligned to the TM-1 *G. hirsutum* reference genome [43] (Table S2). To determine the normal transcriptome differences between the two cultivars, uninfected MCU-5 roots (0 hpi) were directly compared against Siokra 1–4 roots at 0 hpi that identified a large number of differentially expressed genes (DEGs) (15,224) with 6699 DEGs higher expressed in MCU-5 than Siokra 1–4, and 8525 DEGs with lower expression in MCU-5 than Siokra 1–4 (Table S3). When comparing infected to mock-treated root tissue within each cultivar, there were also differences in the number and type of DEGs observed (Table 4). At 6 hpi, the resistant MCU-5 had 411 DEGs (248 upregulated, 163 downregulated) that decreased to 37 (10 upregulated, 27 downregulated) by 1 dpi, and then decreased again to 14 DEGs (5 upregulated, 9 downregulated) at 3 dpi (Table 4 and Tables S4–S6). The susceptible Siokra 1–4 had 204 DEGs at 6 hpi (87 upregulated, 117 downregulated), which decreased to 128 (41 upregulated, 87 downregulated) at 1 dpi but then increased to 671 DEGs (171 upregulated, 500 downregulated) at 3 dpi, (Table 4 and Tables S7–S9). Of the 455 unique DEGs identified between MCU-5 infected and mock-treated roots and the 973 unique DEGs identified between Siokra 1–4 infected and mock-treated roots, only 50 (3.6%) were found to be in common between MCU-5 and Siokra 1–4.

Gene ontology (GO) enrichment analysis (FDR < 0.01) was performed on DEGs from the cultivar comparisons and different infection/mock-treated time points to help classify the type of gene expression pathways altered in the root transcriptomes. The resistant MCU-5 initial infection (6 hpi) was associated with 114 GO terms, 51 biological processes (BP), 37 molecular functions (MF), and 26 cellular components (CC) (Figure S3 and Table S10) that included the response to abiotic stimuli, stress, lignin metabolic and biosynthetic processes, and phenylpropanoid biosynthesis. At 1 dpi, MCU-5 infection was associated with three GO terms (all BP, Figure S4 and Table S11), response to heat, temperature stimuli, and protein folding, and at 3 dpi, twenty-eight GO terms (26 BP and 2 MF, Figure S5 and Table S12) that included the response to abiotic stimulus, stress, and regulation of nitrogen compound metabolic processes. For the susceptible Siokra 1–4, ninety-one GO terms were associated at 6 hpi (66 BP, 22 MF, and 3 CC, Figure S6 and Table S13) including stress responses associated with water deprivation, wounding, and defense response. At 1 dpi, sixteen GO terms (8 BP, 2 MF, and 6 CC, Figure S7 and Table S14) were associated with responses to stress, heat, temperature stimuli, cadmium ion, and cell wall. At 3 dpi, ninety GO terms (42 BP, 14 MF, and 34 CXC, Figure S8 and Table S15) were mostly associated with stress, including light and cadmium ion, changes in the endomembrane system and organelle membrane and lignin and phenylpropanoid biosynthetic processes.

### 2.4. Potential Candidate Genes Associated with VW Resistance

To identify potential candidate genes associated with VW resistance in MCU-5, the list of DEGs found either between uninfected MCU-5 and Siokra 1–4 roots (0 hpi), or between infected and mock-treated MCU-5 at 6 hpi, 1 dpi, and 3 dpi, was crosschecked against the genomic regions (LD 205, LD206, LD207, and LD333) where resistance was mapped in the 240 F_7_ RILs. Ninety-nine DEGs from the 0 hpi cultivar comparisons were located in the LDs associated with VW resistance (Table S16). One of them, Ghi_D09G09601 (carboxylate clamp-tetratricopeptide repeat (TPR) proteins), responded significantly to *V. dahliae* infection at 1 dpi in both MCU-5 and Siokra 1–4 (Tables S5 and S8).

## 3. Discussion

VW is an economically important disease of cotton worldwide, but unlike most cotton-growing countries, in Australia the ND VW pathotype is widespread and highly virulent. The prevalence of VW is increasing in Australia despite growing cultivars with relatively high levels of VW resistance [6]. This increase may be associated with the frequent irrigation and high nitrogen nature of the Australian cotton industry, the spread and increased incidence of exotic diseases such as Black Root Rot [26,44] that can damage cotton roots and may enable *V. dahliae* to colonize weakened plants more easily [45], or the widespread growing of resistant varieties may have inadvertently selected for *V. dahliae* strains that are able to avoid host resistance [6]. As VW is now well established in the Australian cotton industry and is difficult to manage, the breeding of new cotton varieties with increased levels of VW resistance is a priority. To accelerate this breeding effort, understanding the genetics and identifying genomic regions associated with plant resistance is critical.

Investigations into the genetics of VW resistance are difficult as disease severity is highly dependent on environmental conditions and disease quantification measurements are often subjective. To minimize some of these variables, our VW assays were performed under controlled conditions, and using an F_7_ RIL population not only simplified the genetic structure of the population by reducing the level of heterozygosity, but also enabled lines to be replicated so that the more quantitative measures of plant resistance, survival, and shoot biomass measurements could be made by directly comparing to mock-infected plants. Australian cotton cultivars are known to become more susceptible to VW disease when soil temperatures drop below 22 °C [6]. The reason for this is currently unknown; however, cotton growth is highly temperature dependent [46,47], and root growth is much reduced at temperatures around 20 °C [48]. Fusarium wilt (FW) was also found to be more severe in bioassays performed at 23 °C than at 26 °C [49], so low temperatures may generally compromise cotton’s defense mechanism from lower levels of metabolism and growth. The temperature of our VW assays was maintained at a high of 22 °C as it produces very severe symptoms and potentially enables the identification of VW resistance that could operate at relatively low temperatures.

There are currently no cotton varieties immune to VW [37] and few studies on cotton resistance to Australian ND VW pathotypes [17], but the Indian cultivar MCU-5 is known to have relatively high levels of resistance to both VW and FW in Australia based on field evaluations [50]. This cultivar is also thought to have contributed most of the VW resistance present in the cultivar Sicot F-1, which although originally developed for increased FW resistance, has higher VW field resistance as measured by commercial VW ranking [51] than cultivars such as Sicala V-1 and Sicala V-2 that were specifically bred for VW resistance. Siokra 1–4 is very susceptible to both VW and FW [52] and so an earlier generation (F_3_-F_4_) of the same RIL population used in this study, was previously analyzed for FW resistance [53]. As expected, the two parents displayed contrasting levels of resistance to the ND VW pathogen in our environmentally controlled bioassay (Figure 1). The distribution of plant survival in the RIL population revealed a slightly bi-modal appearance with the majority of the lines susceptible to infection (Supplementary Figure S1), indicating that resistance requires the presence of multiple major resistance loci. Shoot weight was used as a measure of VW resistance to help potentially separate lines that merely survived from those that were more tolerant. All lines’ growth was affected by infection as no lines had similar shoot weights (90–100%) to mock-infected controls. However, plant survival as a measure was found to be more heritable (0.58 compared to 0.35) and have a higher genomic prediction accuracy (0.73 compared to 0.36) than shoot weight, and so in this population and assay conditions appears to be a better measure of VW resistance.

QTL analysis using a single-locus approach revealed only two major resistance locations on ChrD03 (LD blocks 205–207) and ChrD09 (LD block 333), and the markers associated with shoot weight and survival in those blocks were similar, with the most significant marker for each region being the same (Chr03_255870 and Chr06_48100923). The multiple-locus QTL approach identified two significant markers for weight and survival that were the same as the most significant markers in the single-locus approach, and Chr03_2558470 (ChrD03) and Chr06_48100923 (ChrD09), explained 0.14 and 0.37 and 0.06 and 0.20 proportion of the phenotype variation for shoot weight and survival, respectively. The QTL results indicate that resistance is a complex trait as only around half of the phenotypic resistance could be explained for survival, but there are two major genomic locations that represent good targets for introgressing into breeding lines using the SNP markers Chr03_2558470 and Chr06_48100923.

Although this study is the first investigation of cotton resistance to an Australian ND *V. dahliae* isolate, there have been many studies that have investigated the genetics of cotton’s response to *V. dahliae* infection [3], although mainly with D VW pathotypes, as worldwide this is the virulent pathotype. A recent meta-analysis of thirty-one VW resistance studies between 2008 and 2022 [54] found QTLs distributed among all cotton chromosomes except five (ChrA02, ChrA04, ChrA09, ChrA13, and ChrD06), highlighting the complexity of VW resistance. Similar to other meta-analysis studies of VW resistance, most QTLs from the different studies were found on ChrD09 with forty [55] and ChrD03 had ten. The meta-analysis by Huo et al. [54] identified a single MQTL on both ChrD03 (MQTL-D03.1) and ChrD09 (MQTL-D09.1), but these do not overlap with the two regions identified in this study. Analysis of the VW resistance of the Upland cotton Prema [56] did identify a major QTL on ChrD09 (qVW-D9-1) between the SSR markers NAU2954-NAU3414 that explained 60.1 to 65.5% of the phenotypic variation observed in an artificial disease nursery. This QTL is present in a similar location to the marker Chr06_48100923, so it is possible that resistance against a Chinese D *V. dahliae* isolate may be the same gene as that against an Australian ND *V. dahliae* isolate, although in our study the ChrD09 locus explains much less of the phenotypic variation than qVW-D9-1, and is also less significant than the D03 region (0.2 to 0.37 PVE) in this study.

The cultivar Sicot F-1 and its parent MCU-5 are highly resistant against both VW and FW, indicating there may be resistance loci present in similar regions between the two diseases. Abdelraheem et al. [57] found a cluster of FW and VW QTL on two chromosomes D05 and D07 but most resistance QTL identified did not co-locate. A previous FW study based on an early generation (F_3_-F_4_) of the MCU-5 x Siokra 1–4 RIL population used in this study [53] did not find QTL in the same location as the VW loci identified in this study, but Wang et al. [58] identified four QTL associated with FW resistance with two, qFW-D3-1 and qFW-D9-1, near the VW regions associated with SNP markers Chr03_2558470 and Chr06_48100923. Liu et al. [59] later went on to identify that the GhGLR4.8 gene confers resistance to *Fov* race 7 in Upland cotton in qFW-D3-1 which is located in LG 207 from this study. So, it is possible that the selection for FW resistance may have also fortuitously carried along VW resistance.

A transcriptome analysis of roots taken from infected and uninfected MCU-5 and Siokra 1–4 plants was performed to help understand the molecular basis of VW resistance and potentially identify candidate resistance genes. Early time points in the infection process were chosen to avoid responses associated with diseased tissue, especially with the susceptible Siokra 1–4. Transcriptome analysis of roots taken before *V. dahliae* inoculation (0 hpi) revealed that there were very large transcriptional differences between MCU-5 and Siokra 1–4 (15,224 DEGs) that were an order of magnitude larger than the differences observed between infected and mock-treated roots from the same cultivar, possibly reflecting the large genetic dissimilarity of these two lines. MCU-5 had a relatively large number of DEGs early after infection (6 hpi) with slightly more genes upregulated than downregulated (248 versus 163) associated with responses to stress, and known VW defense mechanisms associated with lignin and phenylpropanoid biosynthesis [60]. The number of DEGs associated with infection in MCU-5 then declined with more downregulated than upregulated, until by 3 dpi there were only fourteen that were associated with abiotic stress and regulation of nitrogen compound metabolic processes. In contrast, except for the 6 hpi response, Siokra 1–4 had more DEGs than MCU-5 with the majority resulting in downregulation of gene expression. For Siokra 1–4, DEGs were associated with stresses such as water deprivation, and wounding but the genes associated with the defense mechanisms associated with lignin and phenylpropanoid biosynthetic processes were not evident until 3 dpi. Only 3.6% (fifty) of the unique DEGs from the time series were found to be in common between MCU-5 and Siokra 1–4, highlighting the different transcriptional responses of these two cultivars. Previous transcriptome and cytological investigations comparing resistant and susceptible cotton varieties to VW have been performed [59,60,61,62,63,64,65] and found that resistant lines often contain more terpenoids and phenolics than susceptible varieties that are detected earlier in roots of the resistant as compared to the susceptible line. Guo et al. [66] found that the expression of an ethylene response-related factor (*GbERF1*) improved VW resistance in cotton via activation of lignin synthesis. So, it is possible that MCU-5 is better able to resist VW infection due to an earlier defense response mounted compared to Siokra 1–4.

The 382 annotated genes (Table S19) that are located in LD 205, LD206, LD207, and LD333 are candidate genes for the MCU-5-associated VW resistance. The transcriptome experiment identified 99 DEGs that were located in these LD regions. Ghi_D09G09601, a carboxylate clamp-TPR gene, was differentially expressed from the MCU-5 time course but was also differentially expressed in the uninfected root MCU-5/Siokra 1–4 comparison. Therefore, all of the potential candidates identified the four LD blocks were significantly differentially expressed between the cultivars before the roots were infected, indicating that resistance may result from constitutive expression differences between the two cultivars. Among the 99 DEG are three putative disease resistance genes, Ghi_D03G01221, Ghi_D09G09736, and Ghi_D09G09866, that may represent good candidates as these types of genes have been previously associated with resistance to VW in cotton [67,68,69,70], although VW resistance has been associated with genes that are not classical *NBS-LRR* resistance genes [67,71,72,73,74,75,76].

## 4. Materials and Methods

### 4.1. Plant Materials

*Gossypium hirsutum* cv. MCU-5 and *G. hirsutum* cv. Siokra 1–4 were obtained from CSIRO Cotton Breeding, Narrabri, NSW, Australia. MCU-5 is an extra-long staple *G. hirsutum* Indian cultivar [77] derived from a multi-line cross between Indian Coimbatore-type cultivars (MCU-1 and MCU-2) and cultivars from East Africa, the West Indies and the USA, including some contributions from *G. barbadense* cotton. Originally identified to have a high level of resistance to FW [50,53], it was later found to possess high levels of VW resistance. Siokra 1–4 is a VW-susceptible Australian okra leaf *G. hirsutum* cultivar suited to dryland cotton production [50]. An F_7_ population of 240 RIL individuals was originally derived from the F_4_ population studied by Lopez-Lavalle [53] and through single seed descent developed further into an F_7_ population that was previously described and analyzed by Zhu et al. [39] for leaf shape, leaf trichome density, and pollen color. Due to fertility issues, only 240 of the original 244 F_7_ RIL population were used in this study.

### 4.2. V. dahliae Pathotype, Inoculum Preparation and Inoculation for Genetic Mapping and Transcriptome Analysis

An ND VCG 2A, Race 1 *V. dahliae* isolate recovered from a cotton plant displaying severe VW symptoms in a field at the Australian Cotton Research Institute Narrabri (NSW, Australia) was used for all VW infection studies [78]. The growth of the fungus and inoculation procedures were performed as described by Zhu et al. [78]. In brief, the *V. dahliae* isolate was cultured in half-strength potato dextrose broth (12 g/L) for 7 days (25 °C on a shaker, 180 rpm) and the spore concentration of the inoculation solution was adjusted to 1 × 10^7^ conidia/mL. The growth and infection of plants for both the mapping and transcriptome experiments were performed in the controlled environment of a greenhouse with a daytime temperature of 22 °C ± 2 °C with natural lighting and a night temperature of 18 °C ± 2 °C. Inoculation was performed by root dipping by submerging the roots of cotton seedlings (with two true leaves) into the *V. dahliae* solution for 5 min and then transplanting them into the soil (60:40 mix of compost and perlite) in 8 cm pots. Seedlings that acted as controls for mock infection were treated the same except that they were dipped in sterile water. Seedlings of parental lines MCU-5 and Siokra 1–4 were used as controls. The assays on each RIL line were performed independently at least twice with each individual assay containing three technical replicates (5 seedlings per replicate, 15 plants in total) of each line infected and the exact same number of mock-infected replicates. Disease severity of seedlings was evaluated using one of two methods, the percentage of plants alive after four weeks compared to the mock-infected plants of the same line, or the % of fresh weight of the shoot tissue from each infected replicate plant compared to the shoot fresh weight of mock-infected plants from the same line.

For the transcriptome experiment, roots were collected at 6 hpi, 1 and 3 dpi from VCG 2A, Race 1 *V. dahliae* inoculated (infected samples), and water-treated (mock samples) seedlings of MCU-5 and Siokra 1–4. Three biological samples were taken for each treatment (both pathogen-infected and mock-treated). Each sample included roots from three seedlings. The collected samples were immediately frozen in liquid nitrogen and stored at −80 °C for RNA isolation. In addition, three root samples (biological replicates) were collected from untreated seedlings of MCU-5 and Siokra 1–4 for transcriptome sequencing to compare the basal transcriptome difference of the two accessions and their association with the difference in basal disease tolerance. 

### 4.3. Plant DNA Sample Preparation and Genotyping

The DNA of all *G. hirsutum* lines was extracted from young leaves according to the method described by Ellis et al. [79]. DNA quantity was measured using the NanoDrop ND-1000 spectrophotometer (NanoDrop Technologies, Wilmington, DE, USA) and adjusted to a working concentration of 20 ng/µL. 

Genotyping of the F_7_ RIL MCU-5 x Siokra 1–4 population and parental lines using a custom SNP Illumina GoldenGate SNP assay that was performed by Beijing Genomics Institute (BGI Hong Kong, China) was previously reported in Zhu et al. [39]. This 1308 GoldenGate SNP dataset (SNP markers named according to their positions determined from the D5 genome of *G. raimondii*) [40] was supplemented with 29 SSR markers (Supplementary Table S17) that were performed as previously described [79]. In total, data were obtained on 1337 polymorphic markers that were mapped to the genetic standard line TM-1 from Wuhan University, Wuhan, China (WHU) [43].

### 4.4. Linkage Disequilibrium (LD) Analysis

The LD network approach [41] was used to cluster 1337 markers into LD blocks (Supplementary Table S18). In total, the markers were classified into 461 LD blocks, with 2.9 markers as the average number of markers in each LD block. The majority of LD blocks (249) only included a single marker, whereas 21 LD blocks had more than 10 markers, with the largest LD block (#317) found on ChrD08 with 28 markers.

### 4.5. Quantitative Trait Locus (QTL) Mapping

Both the percentage shoot weight and percentage plant survival values were averaged across the two biological replicates, and the averaged values were considered phenotypes in the QTL mapping. Two QTL mapping methods were applied: a single-locus mapping using linear regression and permutation test [41], and multiple-locus mapping using a modified Bayesian stochastic search variable selection (SSVS) algorithm which incorporated the LD information as a model prior [42]. The single-locus approach analyzed one marker at a time and estimated the marginal genetic effect of each marker using standard linear regression. A permutation test was then used for multiple testing, and to formally judge QTL. This single-locus approach has been the most widely applied method in the plant and animal genetics community. This approach was implemented using the R code available at [41]. In contrast, the multiple-locus approach analyzed all the markers simultaneously and estimated the conditional genetic effect of the markers, which can be helpful to more accurately locate the QTL region and control the false positives [41]. Here, we used a Bayesian SSVS regression approach [42] which assigned a spike and slab prior to the genetic effects of each SNP, which is used to select only a subset of SNPs that are associated with the phenotypes in the model and discard the unimportant ones. When incorporating the LD information in the prior, this new approach has been proven to have better power to detect QTL, compared to other Bayesian regression methods such as Bayes C [80]. In practice, this Bayesian multiple-locus approach was implemented using the R code available from Li et al. [42]. Genomic heritability, or the proportion of the phenotype variance explained by all the markers, of the percentage shoot weight and percentage plant survival traits was estimated using the multiple-locus approach.

### 4.6. Genomic Prediction

The predictive power of the multiple-locus method on the weight and survival traits was evaluated using a 5-fold cross-validation strategy. The data (240 samples) were randomly divided into 5 parts with equivalent sample sizes. In turn, each part (having 48 samples) was used as the test population, and the rest of the 192 samples were used as the training population. The prediction accuracy was measured by Pearson correlation between genomic estimated breeding values and true phenotypes of the test population.

### 4.7. Total RNA Extraction and Transcriptome Sequencing

Total RNA of whole root samples was extracted using the RNeasy Plant Mini Kit (Qiagen, Hilden, Germany) by following the manufacturer’s instructions. After checking the quality and integrity of RNA using the Agilent 2100 Bioanalyzer (Agilent, Santa Clara, CA, USA), 5 µg of total RNA per sample was submitted to the Australian Genome Research Facility (AGRF, Melbourne, VIC, Australia) for transcriptome sequencing, which was performed using the paired-end (150 bp) configuration on an Illumina HiSeq 2000 instrument (Illumina, San Diego, California, USA) according to the manufacturer’s instructions. Approximately 8 Gb of data were generated for each sample. Raw reads were first processed using Trimmomatic v0.39 [81] to remove low-quality sequences and adaptors. The quality of trimmed FASTQ files was evaluated using FastQC v0.11.8 [82]. Reads were mapped to the *G. hirsutum* genome of the genetic standard line TM-1 from Wuhan University, Wuhan, China (WHU) [43] using STAR v2.7.9a [83], and transcript per million mapped reads (TPM) was calculated for estimating gene expression levels with a custom Python script. Counts were obtained with htseq-count [84] with Python v3.9.4. The differential gene expression calculation was performed using DESeq2 v1.30.1 [85] in R v4.0.5 [86] and transcripts with Bonferroni Hochberg adjusted *p*-values of <0.05 were considered DEG. DEG fold-change values are always presented as infected/mock-treated. The raw RNA-Seq data are available from the CSIRO data portal (https://doi.org/10.25919/41ab-xc19, accessed on 12 February 2024). Gene ontology (GO) enrichment analysis was performed using agriGO v2.0 based on the default settings [87] and only terms with a false discovery rate (FDR) of <0.01 were selected as significant. The gene lists for GO analysis were obtained by finding DEGs of infected versus control samples at time points 6 hpi, 1 dpi, and 3 dpi.

## 5. Conclusions

The VW resistance of MCU-5 to a virulent Australian ND VCG2A *V. dahliae* was found to be complex, but the two major genomic locations identified represent good targets for introgressing additional levels of resistance into Australian breeding lines. A combination of genetic mapping and transcriptome analysis was able to identify a number of potential candidate resistance genes for further investigation. In the future, gene editing will be used on potential candidate genes to determine which are important for this VW resistance.

## Figures and Tables

**Table 1:** Results of QTL mapping based on relative shoot weight using the single-locus method.

Marker ID ^1^	Chr	Position (bp)	LD Block	*p*-Value ^3^
Chr03_1555481	D03	1706465	206	0.03
Chr03_1763859	D03	1939003	206	8 × 10^−4^
Chr03_2557996	D03	2640629	207	0.02
**Chr03_2558470 ^2^**	**D03**	**2641126**	**207**	**3 × 10^−5^**
Chr03_3891990	D03	3965470	205	6 × 10^−4^
Chr03_4130001	D03	4206876	205	3 × 10^−4^
Chr03_4841415	D03	4972829	205	5 × 10^−4^
Chr03_6732381	D03	14128895	205	0.03
Chr06_47522483	D09	46353562	333	3 × 10^−4^
Chr06_47412227	D09	46205935	333	0.01
**Chr06_48100923**	**D09**	**47006202**	**333**	**7 × 10^−5^**
CGR6806	D09	47149770	333	1 × 10^−4^

^1^ Markers are ordered based on chromosome and position on *G. hirsutum*, with their IDs designated using *G. raimondii* chromosome and position. ^2^ Markers in bold are the most significant in each Chr location. ^3^ *p*-values < 0.05 were considered significant.

**Table 2:** Results of QTL mapping based on plant survival using the single-locus method.

Marker ID ^1^	Chr	Position (bp)	LD Block	*p*-Value ^3^
Chr03_1241169	D03	1319099	206	0.03
Chr03_1555481	D03	1706465	206	6 × 10^−5^
Chr03_1504732	D03	1619794	206	5 × 10^−5^
Chr03_1763859	D03	1939003	206	1 × 10^−6^
Chr03_2277315	D03	2318366	207	3 × 10^−8^
Chr03_2557996	D03	2640629	207	3 × 10^−10^
**Chr03_2558470 ^2^**	**D03**	**2641126**	**206**	**3 × 10^−19^**
Chr03_3526626	D03	3575466	205	3 × 10^−4^
Chr03_4130001	D03	4206876	205	9 × 10^−7^
Chr03_4841415	D03	4972829	205	2 × 10^−6^
Chr03_6732381	D03	14128895	205	2 × 10^−3^
Chr06_47522483	D09	46353562	333	1 × 10^−5^
Chr06_47412227	D09	46205935	333	0.01
**Chr06_48100923**	**D09**	**47006202**	**333**	**2 × 10^−7^**
CGR6806	D09	47149770	333	2 × 10^−6^
Chr06_47820414	D09	46664265	333	0.02
Chr06_48139722	D09	47045367	333	0.01
Chr06_48729925	D09	47645669	332	0.01

^1^ Markers are ordered based on chromosome and position on *G. hirsutum*, with their IDs designated using *G. raimondii* chromosome and position. ^2^ Markers in bold are the most significant in each Chr location. ^3^ *p*-values < 0.05 were considered significant.

**Table 3:** Results of QTL mapping using multiple-locus method.

Trait	Marker ID	Chr	Position (bp)	LD Block	Selection Probability	PVE *
Weight	Chr03_2558470	D03	2641126	207	0.99	0.14
	Chr06_48100923	D09	47006202	333	0.66	0.06
Survival	Chr03_2558470	D03	2641126	207	1.00	0.37
	Chr06_48100923	D09	47006202	333	1.00	0.20

* Phenotypic variation explained.

**Table 4:** Number of DEGs between *V. dahliae* infected and mock-treated root tissue of MCU-5 and Siokra 1–4.

	MCU-5		Siokra 1–4	
Time Point	Upregulated	Downregulated	Upregulated	Downregulated
6 hpi	248	163	87	117
1 dpi	10	27	41	87
3 dpi	5	9	171	500

## Data Availability

The raw paired-end Illumina RNA sequencing reads generated in the current study are available from the CSIRO data portal https://doi.org/10.25919/41ab-xc19, accessed on 12 February 2024.

## Supplementary Materials

The following supporting information can be downloaded at https://www.mdpi.com/article/10.3390/ijms25042439/s1.
