# Supplementary material for: Genetic Mapping and Characterization of Verticillium Wilt Resistance in a Recombinant Inbred Population of Upland Cotton
Source: Int J Mol Sci. 2024 Feb 19;25(4):2439. doi: 10.3390/ijms25042439 (PMC10889826; doi:10.3390/ijms25042439)
Supplement: Supplementary file 1 [file ijms-25-02439-s001.zip › Supplementary Figures.pptx]

## Slide 1
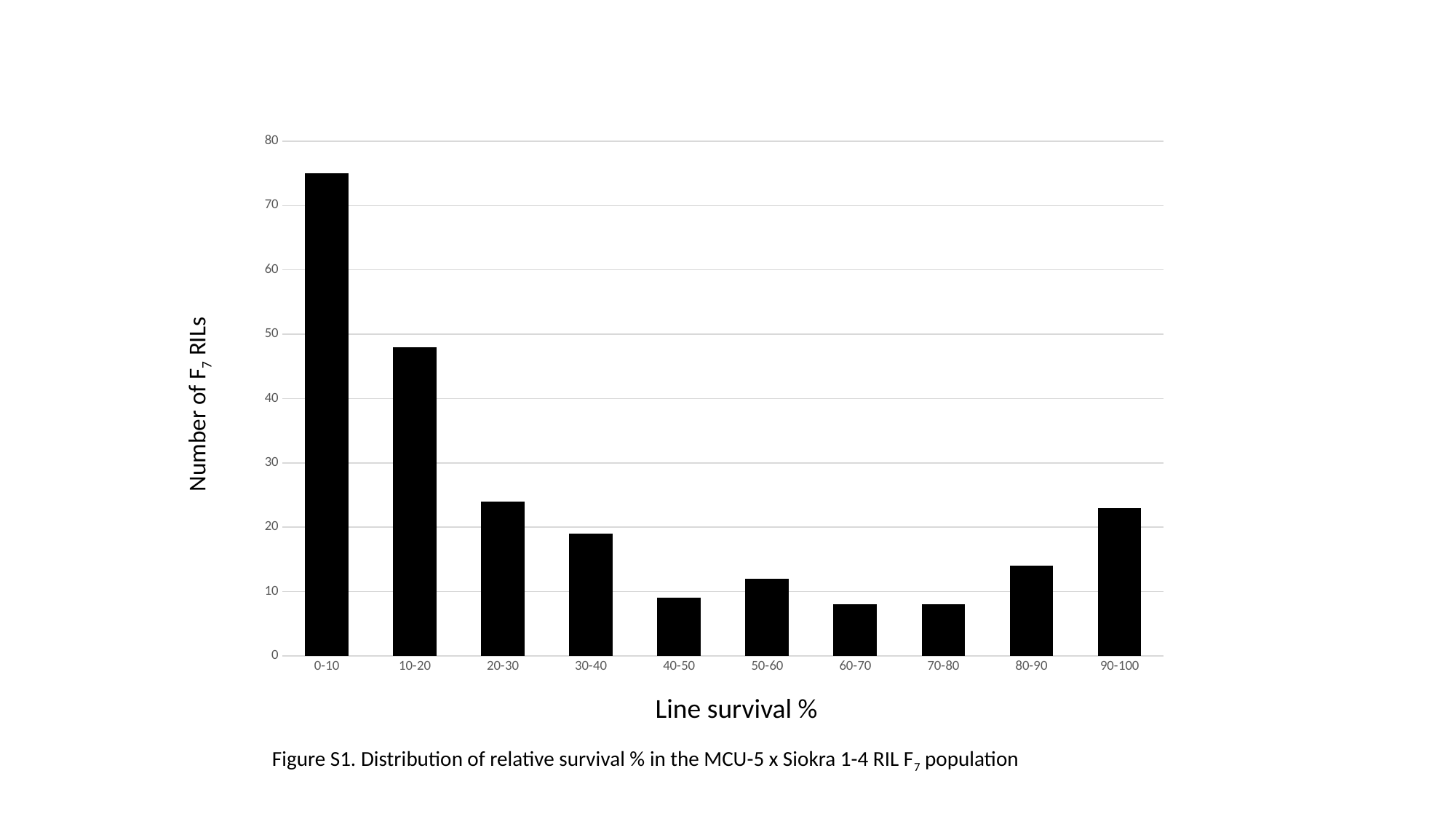

### Chart
| Category | |
|---|---|
| 0-10 | 75.0 |
| 10-20 | 48.0 |
| 20-30 | 24.0 |
| 30-40 | 19.0 |
| 40-50 | 9.0 |
| 50-60 | 12.0 |
| 60-70 | 8.0 |
| 70-80 | 8.0 |
| 80-90 | 14.0 |
| 90-100 | 23.0 |Number of F7 RILs
Line survival %
Figure S1. Distribution of relative survival % in the MCU-5 x Siokra 1-4 RIL F7 population

## Slide 2
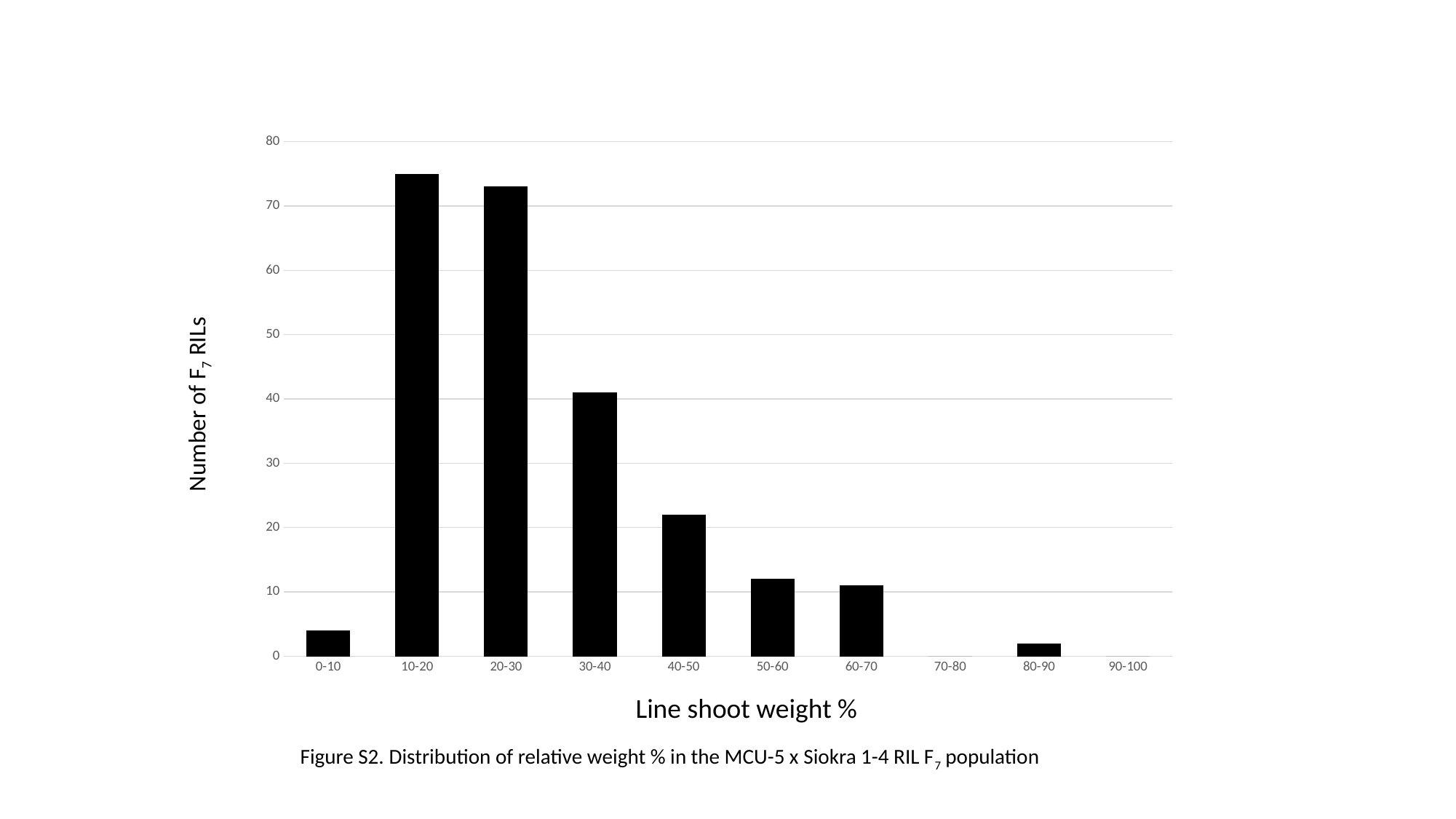

### Chart
| Category | |
|---|---|
| 0-10 | 4.0 |
| 10-20 | 75.0 |
| 20-30 | 73.0 |
| 30-40 | 41.0 |
| 40-50 | 22.0 |
| 50-60 | 12.0 |
| 60-70 | 11.0 |
| 70-80 | 0.0 |
| 80-90 | 2.0 |
| 90-100 | 0.0 |Number of F7 RILs
Line shoot weight %
Figure S2. Distribution of relative weight % in the MCU-5 x Siokra 1-4 RIL F7 population

## Slide 3
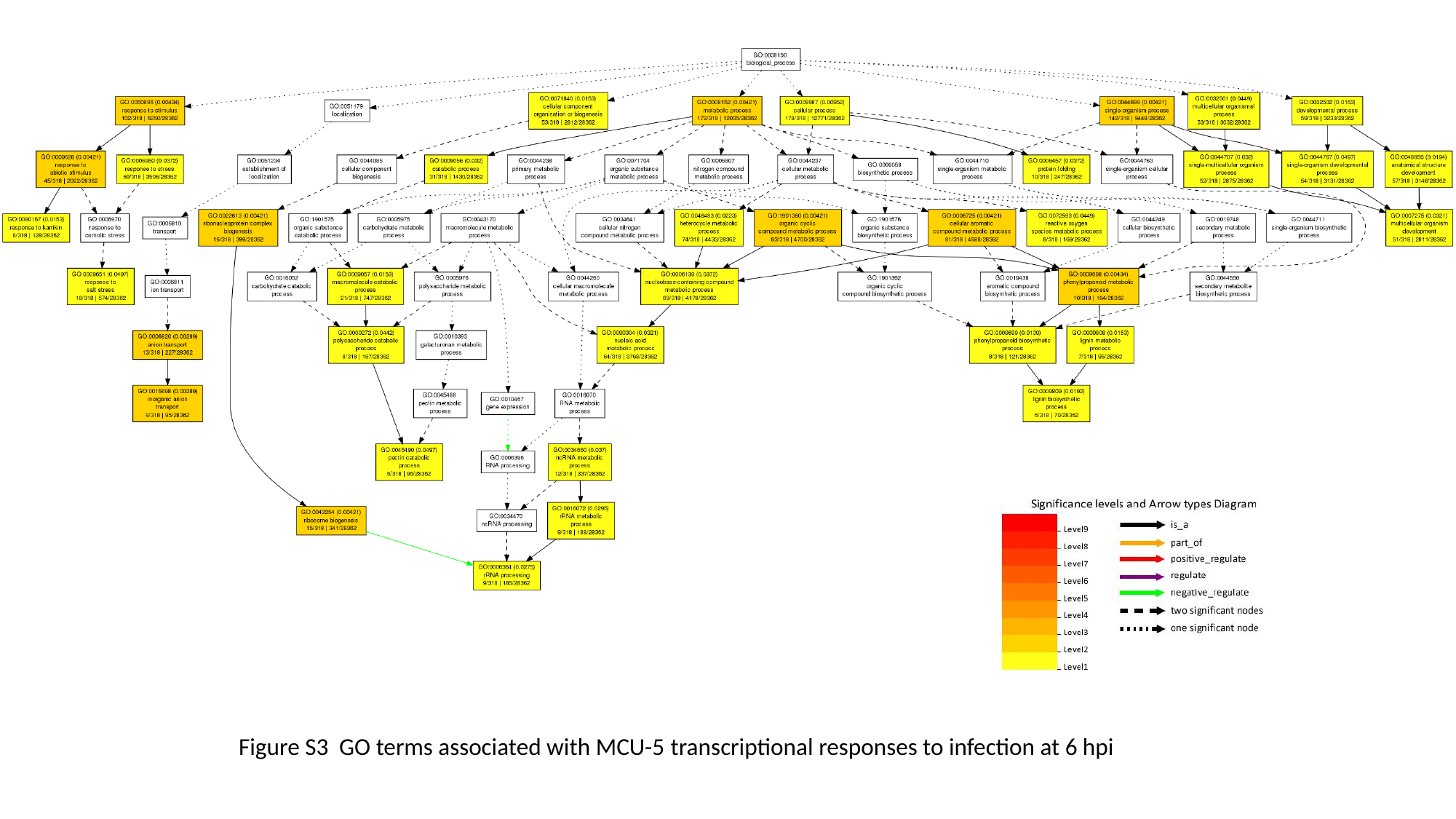

Figure S3 GO terms associated with MCU-5 transcriptional responses to infection at 6 hpi

## Slide 4
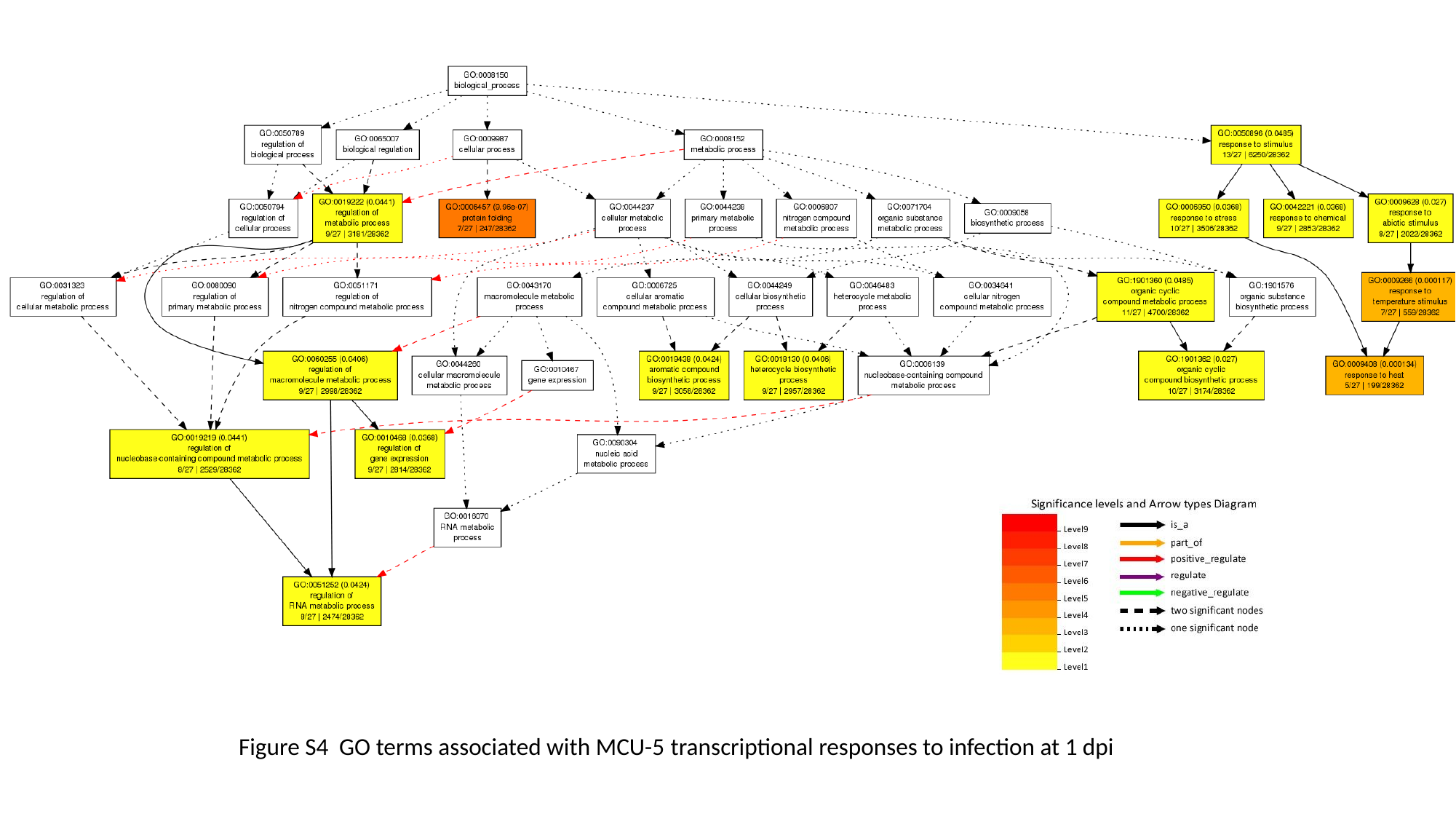

Figure S4 GO terms associated with MCU-5 transcriptional responses to infection at 1 dpi

## Slide 5
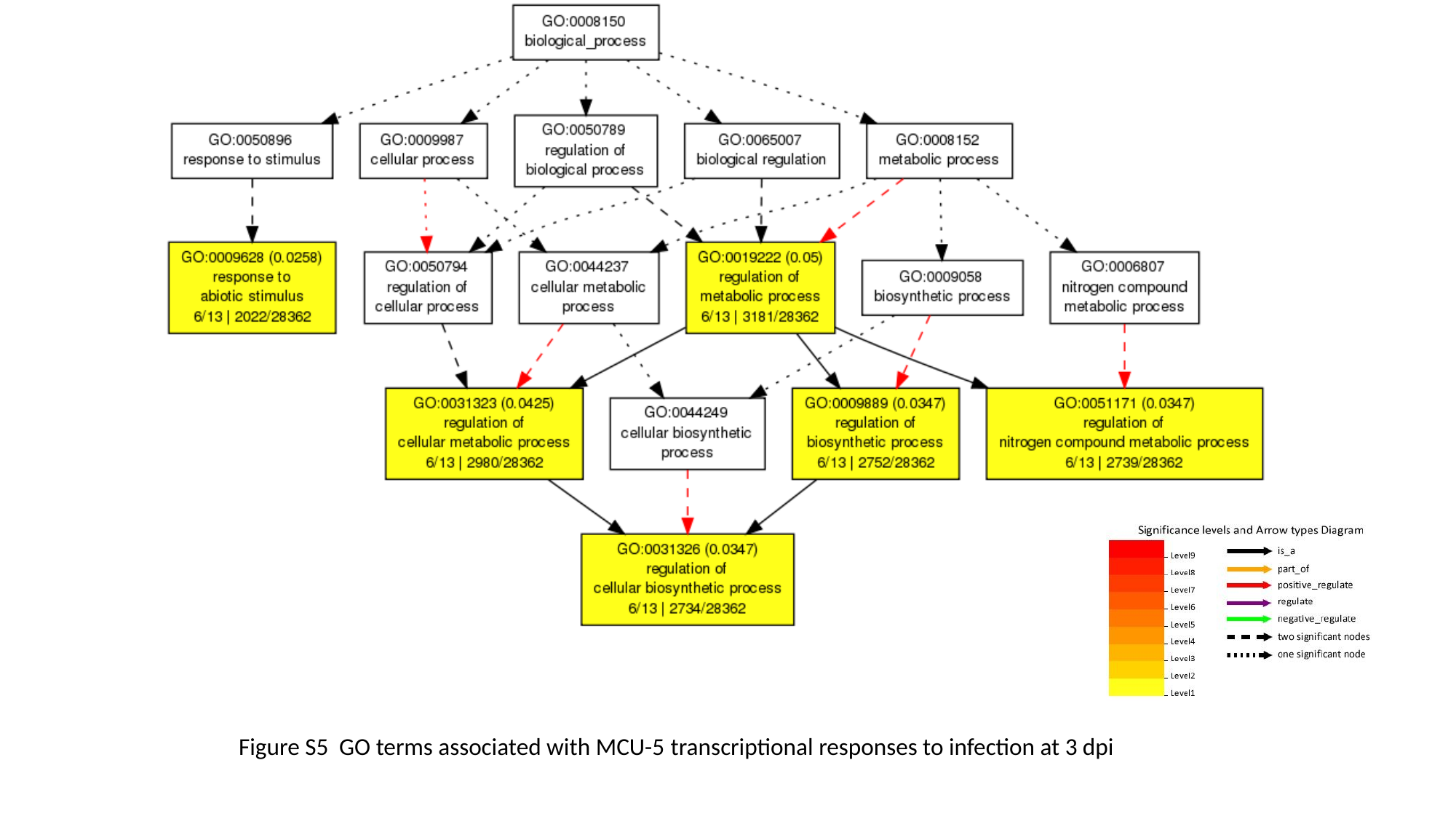

Figure S5 GO terms associated with MCU-5 transcriptional responses to infection at 3 dpi

## Slide 6
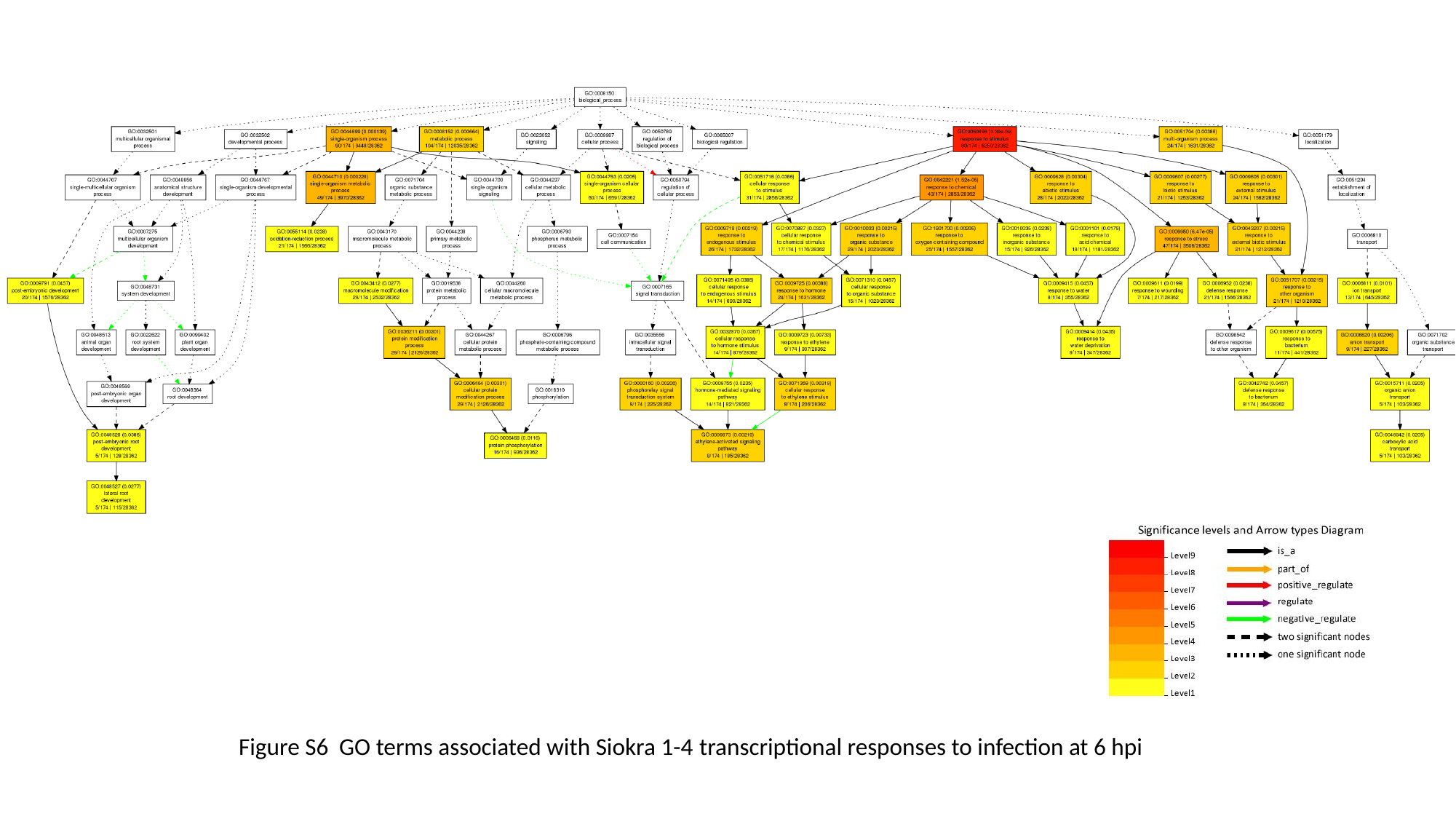

Figure S6 GO terms associated with Siokra 1-4 transcriptional responses to infection at 6 hpi

## Slide 7
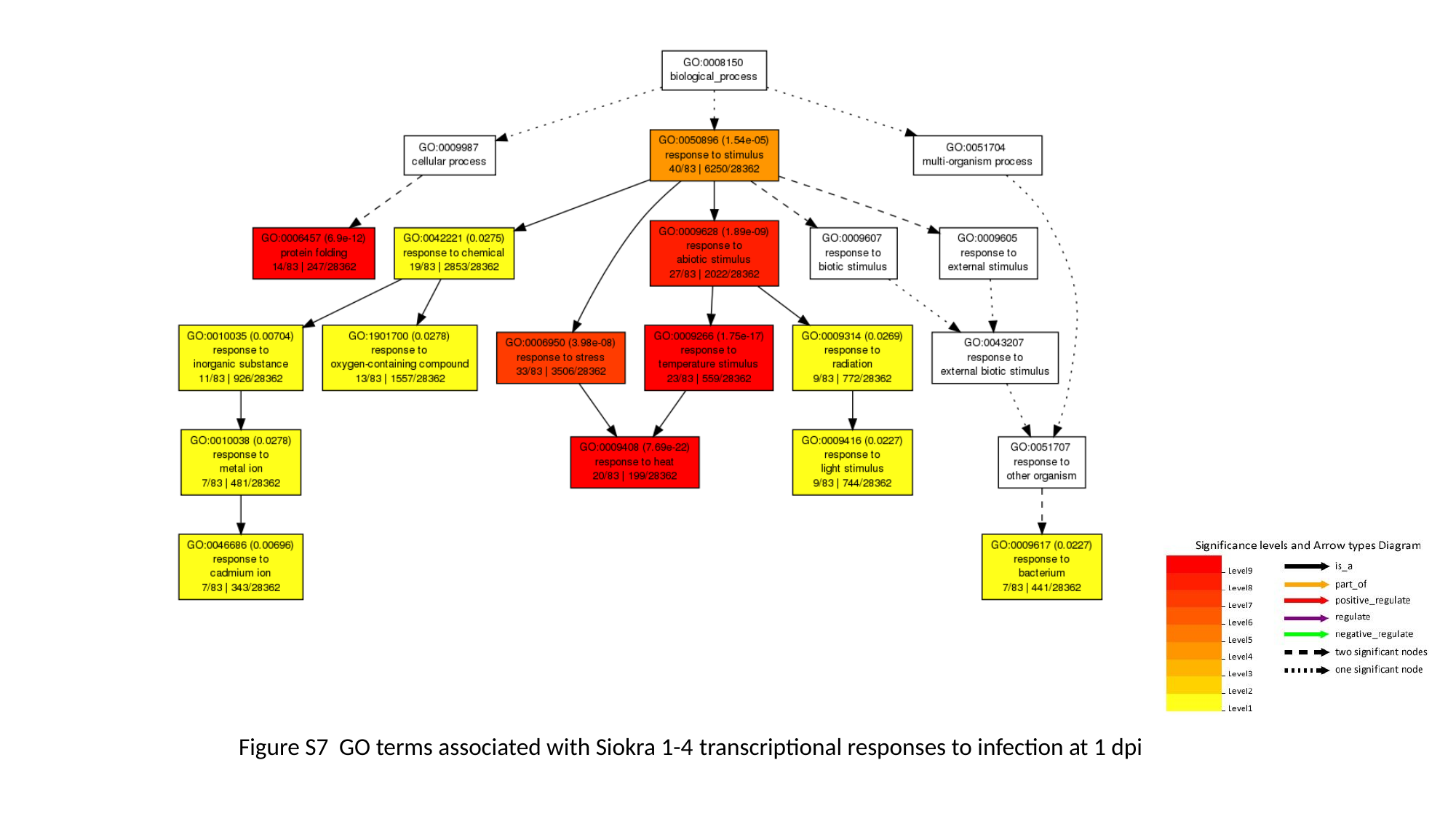

Figure S7 GO terms associated with Siokra 1-4 transcriptional responses to infection at 1 dpi

## Slide 8
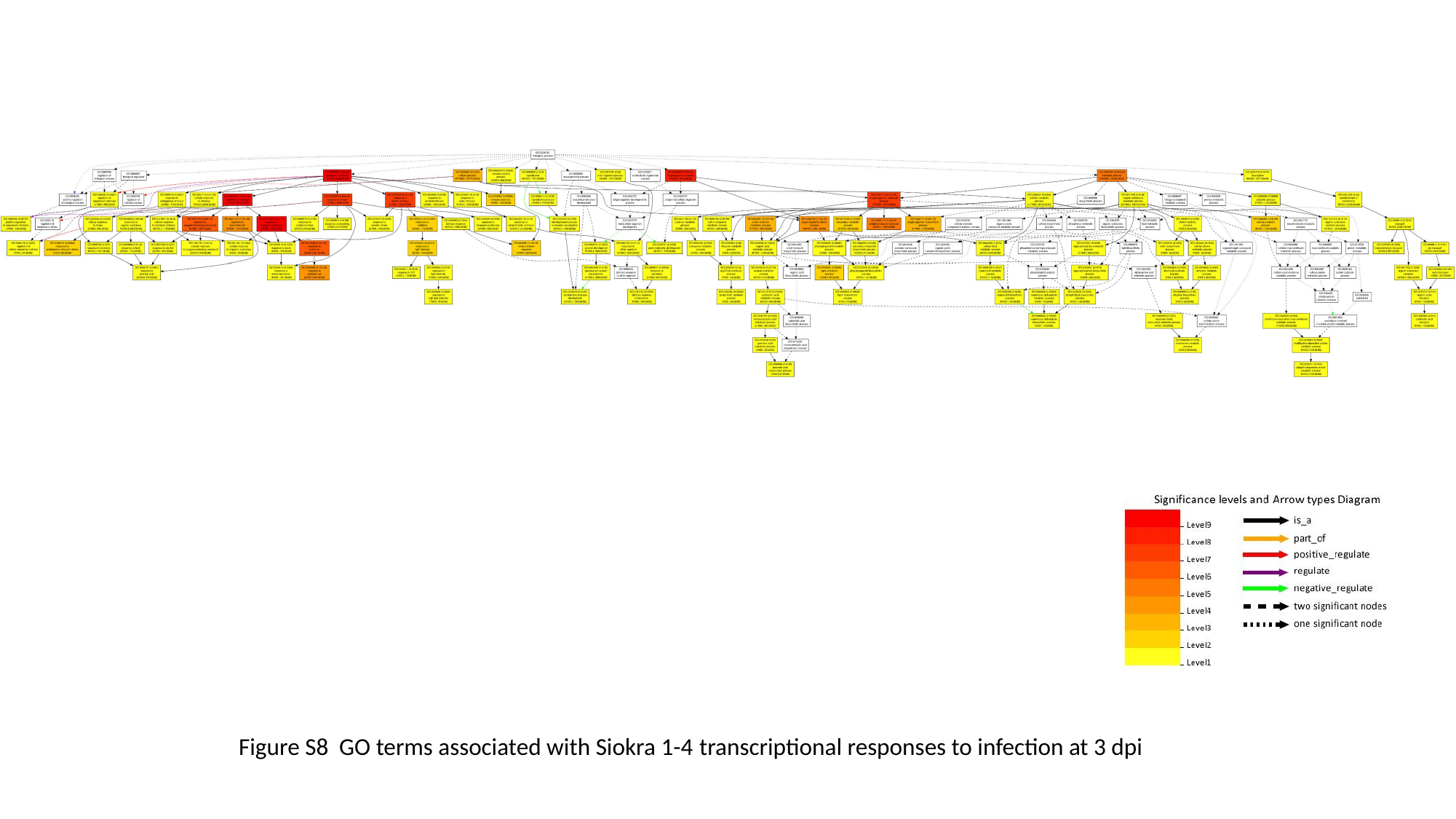

Figure S8 GO terms associated with Siokra 1-4 transcriptional responses to infection at 3 dpi
